# Probing adsorption on a nanoscale: field desorption microspectroscopy

**DOI:** 10.1007/s10450-016-9824-7

**Published:** 2016-10-07

**Authors:** Yuri Suchorski

**Affiliations:** Institut für Materialchemie, Technische Universität Wien, Getreidemarkt 9, 1060 Vienna, Austria

**Keywords:** Field desorption, Field ion microscopy, Field ion appearance energy spectroscopy, Coadsorption, Carbon monoxide, Lithium

## Abstract

Combining an energy analyzer with a field ion microscope equipped with a probe-hole which corresponds to just few atomic surface sites, spatially resolved energy analysis of ions field desorbed from the adsorbent surface is possible on a nm-scale. The experimentally measured values of the kinetic energy of field ions can be related (by means of a thermionic cycle) to the physically meaningful binding energy of corresponding adsorbed species. The development of the technique into a full serviceable micro-spectroscopy on a nanoscale allowed recent detection of the weakly adsorbed CO species on Pt(111) which are largely analogous to those adsorbed at high pressures and provided first results for the binding energy of Li adatoms in a coadsorption system, namely Li–O–W(112) for various lithium and oxygen coverages. In the present contribution, an overview of the experimental possibilities of the technique is given and recent results are discussed.

## Introduction

The term “microspectroscopy” is usually associated with a technique, where an X-ray or UV photon beam is focused into a fine spot and the energy of the locally emitted photoelectrons provides chemical information. By scanning the specimen with this spot, an image with chemical contrast can be achieved (Merkel et al. 2001). The high brightness of modern synchrotron radiation sources allows chemical surface imaging with resolutions in the 10 nm range (Ziethen et al. 2000). This is still far away from the ultimate chemical resolution, that is e.g. achieved with atom-probe (AP) techniques, where single atoms evaporated from the surface layer by high field (or laser) pulses are analysed (Suchorski and Drachsel 2011; Miller and Forbes 2014). However, the AP-approach aims at the 3D-analysis of the specimen (3D-tomography) rather than the adsorbed species on the surface and does not provide, at least in the usual commercial implementation, the binding energy of adsorbed molecules or atoms.^1^

However, in the last decades the demand for methods allowing the study of adsorption on the nm-scale scale has increased, due to fast widening of the assortment of available architectures of nanosized materials, such as nanoparticles, nanorods and nanosheets (Liu et al. 2013). The peculiarities of these novel nanoscale architectures, such as varying contribution of atoms located on confining surfaces of such nanosized material make such systems much more interesting, but additionally increase the difficulties of experimental studies. Even the mere imaging of the nm-scale pore arrangements appears difficult, without speaking about the in situ visualization of dynamic processes occurring in such systems. Although the local scanning techniques, such as STM and AFM allow real time observations of processes in an Angström range (Bliem et al. 2015), the studies of spatial correlation of events as well as the determination of the chemical identity of adsorbed species on an atomic scale or measuring of their binding energy still remain challenging.

**Fig. 1:**
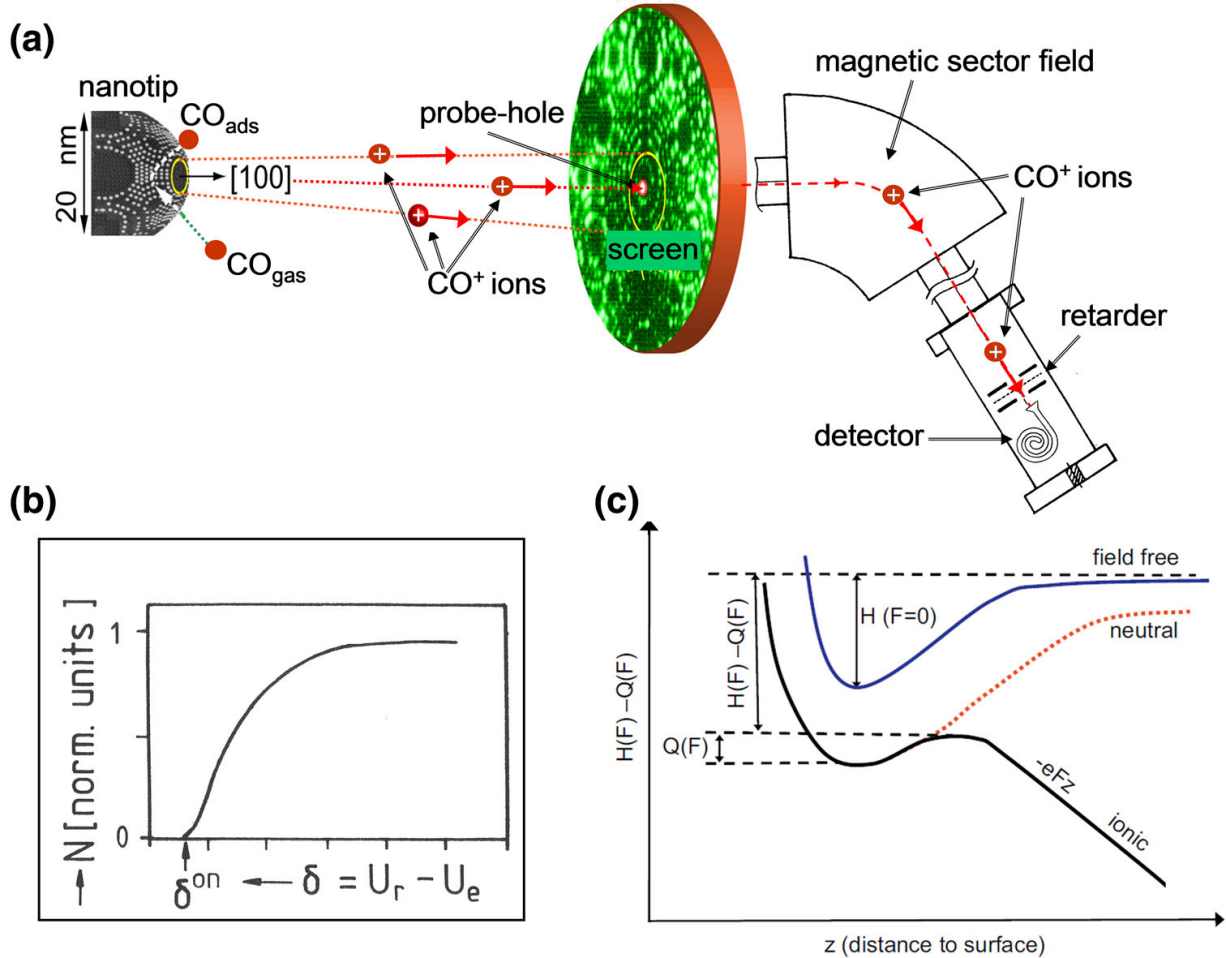
Local mass-to-charge resolved retarding potential analysis of field desorbed species and determination of binding energies: **a** Scheme of the experimental set-up. The nanotip surface covered by adsorbed CO (as an example) is visualized in an FIM by CO^+^ ions desorbed from the surface by high electric field. The CO^+^ ions, which pass the probe hole in the middle of the screen and the magnetic sector field, are analysed in a retarding potential analyser. **b** A typical ion retardation curve, the potential difference U_r_ − U_e_, at which the ions appear first, provides the onset voltage δ^on^. **c** Energy diagram illustrating the energy terms in Eq. (2). The neutral adsorption energy curve is bent by the externally applied field of 10–20 V/nm so that adsorbed species can thermally escape (field-desorb) over the activation energy barrier

In turn, the field ion microscope (FIM), where the imaging ions are radially emitted from the nanotip surface creating an atomically resolved image, allows, due to the parallel imaging principle, the study of spatially correlated effects, such as diffusion (Binh 1983). A combination of an FIM with a small probe-hole in the screen also allows the local sampling of the surface species (Suchorski 2015). The species, field desorbed as ions from the surface, pass the probe-hole, which is adjusted to a few chosen surface sites, and are collected after a time-of-flight or magnetic sector field selection (Fig. 1). From the energy analysis of the selected ions the binding energy of adsorbed species can be determined, as will be explained in detail below. By moving the specimen, the probe-hole “scans” the specimen surface, providing spatially resolved spectroscopic information. Together with the atomically resolved image, simultaneously recorded from the screen, this realizes a *microspectroscopy* on the nm-scale. Since in the case of the magnetic sector field selection, a constant electric field can be used for field desorption, a “gentle” collecting of adsorbed species without damaging the specimen surface is possible.

This concept was first realized experimentally for adsorbed alkali atoms using a lithium field desorption microscope (Li-FDM), where the Li-ions field desorbed from the surface image the latter with a nearly atomic resolution (Medvedev et al. 1994), combined with a retarding potential analyzer. In this way, the binding energy of Li-adatoms field-desorbed from individual surface sites on W(111) was determined (Suchorski et al. 1995; 1996). Since that time, different adsorption systems such as CO/Pt, N_2_/W, O_2_/Pt, etc. were studied (Suchorski 2015). In the present contribution a short overview of our recent studies, namely the results for the weakly bound mobile CO adsorption layers, similar to those that exist during the high pressure adsorption and an example of the coadsorption (lithium and oxygen), are discussed.

## Principles and instrumentation

Although the possibility to visualize individual surface atoms is the most impressive capability of the FIM based techniques, not less important is the ability to analyse the ions of species emitted from the surface by applied electrostatic field. In the case of adsorbed species such process is called *field desorption* and, correspondingly, *field evaporation*, when the atoms of the specimen are emitted. For both processes, the applied field strains the binding between the surface and the atom or molecule going to be emitted, shifting it away from the equilibrium position, till the bond breaks. Therefore, the ions of atoms or molecules removed from the surface, are generated at a small distance *x* (in Angström range) above the specimen surface and have thus an energy which is less than that of ions which would start directly at the surface. The latter would have an energy corresponding to the full accelerating potential of the specimen. The *energy deficit* of an amount *neFx*, where *ne* is the ionic charge and *F* is the field strength near the tip surface, localizes thus precisely the field ionization event. The “bond breaking” distance and the corresponding energy deficit reflect the strength of the binding and thus the binding energy of the adsorbed species. A measurement of the energy deficits of field desorbed ions would then provide information on the *site specific binding energy* of adsorbed atoms or molecules. Energy deficits can be determined from the energy analysis of corresponding field ions: since after the field ionization event the total energy is conserved, the change of the potential energy at the instance of field ionization will contribute to the kinetic energy of the ion in the field-free region.

Of course, the complexity of the necessary instrumentation is much greater than that of conventional FIM, but the degree of the atomistic insight into the adsorption process pays off.

The technique consists of a probe-hole FIM setup combined with an instrument for mass-to-charge-resolved retarding potential analysis of field ions (Fig. 1a). In a probe hole experiment, the adsorbent-specimen (a nanotip, the apex of which is the studied surface) with an adsorbed layer (including multilayer adsorption) is exposed to a high electric field (V/nm range) which modifies the potential curve of adsorbed species (Fig. 1c), lowering the activation energy of desorption *Q*(*F*). At sufficiently low *Q*(*F*), a measurable flux of desorbed ions sets in and the ions (from few chosen surface sites) which pass the probe-hole are mass-to-charge selected in a magnetic sector field and then energy-analyzed with a five-electrode electrostatic retarding potential analyzer. By varying the threshold voltage *δ* = *U*_r_ – *U*_e_ (with *U*_r_ and *U*_e_ being the potentials of the retarder net and of the specimen emitting ions, respectively) the field ion retardation curves *N* = *N*(*δ*) can be obtained for any ions emitted from the selected surface sites on the tip surface (Fig. 1b). From these curves, the *δ* ^*on*^, *onset voltage*, can be determined, as a voltage at which ions just overcome the potential barrier and can be detected. From the onset voltage, the *appearance energy* of field ions, which is by definition the energy required to remove an electron from the neutral species to create a corresponding ground state ion, can be obtained as an empirical value

1 $$A\; = \;n\phi_{\text{ret}} \; - \;ne\delta^{on}$$

with *ϕ*_ret_ being the work function of the retarder (Goldenfeld et al. 1974).

The relation between the appearance energy of field ions and site specific binding energy of adsorbed species was derived theoretically by Forbes from the thermionic cycle long before the experimental realization (Forbes 1976). It provides the relationship between the appearance energy of field-desorbed ions, *A*, their ionization energy, *I*, the field dependent binding energy, *H*(*F*) and the (also field-dependent) activation energy of field desorption, *Q*(*F*):

2 $$A\; = \;I\; + \;H\left( F \right)\; - \;Q\left( F \right)$$

The meaning of the terms *H*(*F*) and *Q*(*F*) is illustrated in Fig. 1c, note that *H*(*F*) is the difference between the minimum of the total energy of the adsorbed atom or molecule in its ground state and the energy of the desorbed free molecule (in field-free space).

For many years, the application of Eqs. (1) and (2) for deriving the binding energies was hindered by problems of in situ determining the absolute work function of the retarder *ϕ*_ret_ and the onset voltage *δ* ^*on*^ simultaneously. While no problems appear with the onset voltage, the in situ work function measurements are complicated. A break-through was achieved in 1993 (Schmidt et al. 1993), by experimentally proving that at transition from localized to non-localized ionization of noble gases, the value of *A* approaches exactly the ionization energy *I*, in full agreement with theoretical predictions (Forbes 1976). This made an in situ calibration of the work function of the retarder possible by substituting *A* in Eq. (1) with the value *I* for a noble probe-gas ionized at elevated temperature (Schmidt et al. 1994).

In the following application examples, Ne and Ar, field ionized at 160 K, i.e. at the temperature where the non-localized ionization takes place, were used for such calibration. Since at 160 K, *A*_Ar,160K_ is equal to *I*_Ar_, the value of *ϕ*_ret_ could be obtainted in situ from Eq. (1). Once *ϕ*_ret_ is known, an absolute determination of the *A* values for any other field-ionized species can be performed (in the same apparatus), including field-desorbed entities. Details of the experimental set-up including the tip assembly, adapted for liquid nitrogen cooling as well as for direct current heating (operating temperature between 79 and 600 K), and the gas-supply systems were described elsewhere (Schmidt et al. 1994). The calibration of the applied electric field is based on the standard values of the evaporation field for corresponding metals (Müller and Tsong 1974).

The development of the technique into a full serviceable micro-spectroscopy (where the probe hole can scan the surface during the simultaneous FIM imaging) on a nanoscale allowed recent detection of the weakly adsorbed CO species on Pt(111), which are largely analogous to those adsorbed at high pressures, and provided first measurements of the binding energy of particular adatoms in a coadsorption system, namely Li–O–W(112) for various lithium and oxygen coverages. These two application examples are reviewed below in detail.

## Application examples

### Weakly adsorbed CO species on Pt(111)

**Fig. 2:**
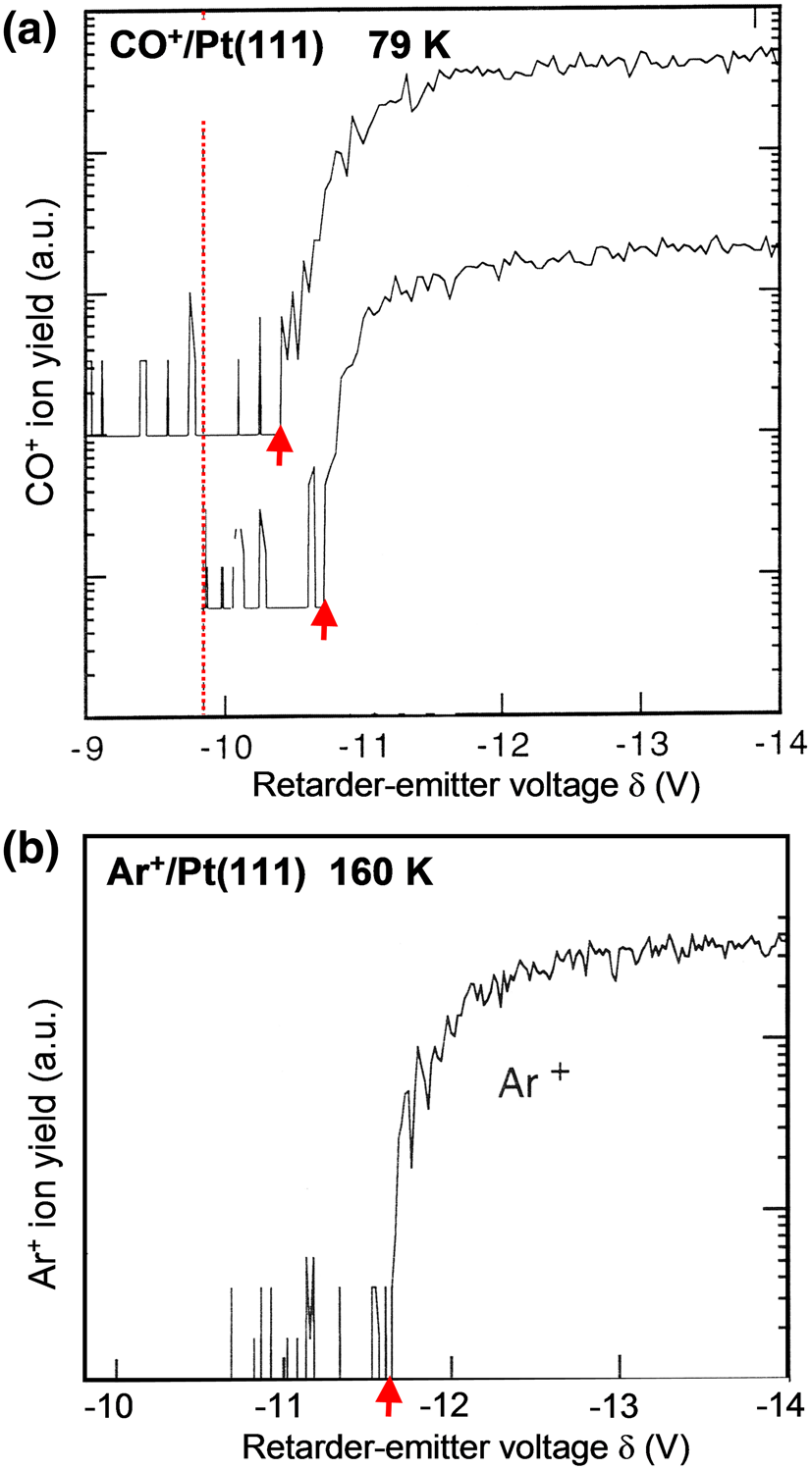
Weakly bound CO species on a Pt(111) nanofacet: **a** Retardation curves for CO^+^ ions emitted from Pt(111) at 15.4 V/nm (*upper curve*) and at 18.9 V/nm (*lower curve*) at 79 K. The dotted line indicates the potential onset position for *A*_CO_ = *I*_CO_. **b** The retardation curve for Ar^+^ ions emitted from the same surfaces sites at 160 K, which was used for calibration of *ϕ*_ret_: the onset value read from the abscissa allows an in situ determination of *ϕ*_ret_ in Eq. (1)

The role of the weakly adsorbed CO species on Pt-catalyst surface, which are assumed to exist at elevated pressures, is often and controversially discussed in the literature (Freund et al. 2011; Farias et al. 2015). The experimental results are rather scarce due to the difficulties in detecting such layers in UHV. High electric field might stabilize such weakly bound layers facilitating the experimental verification. Figure 2 shows typical retardation curves for CO^+^ and Ar^+^ ions emitted from the same few surface sites on the apex facet of a [111]-oriented Pt tip in the applied field range of 15–19 V/nm. The Ar^+^ retardation curve shown on the right was used for the in situ determination of the work function of the retarder, as described above.

Retardation curves for CO^+^ ions shown in (Fig. 2a) exhibit two remarkable features: (i) the onsets are shifted well above the values corresponding to the ionization energy threshold of the free CO molecule (marked by the dashed line) (ii) at increasing field strength the onsets shift to the right, i.e. the appearance energy *A* increases. These observations are in good agreement with earlier findings for other CO/metal systems and are in accord with the field desorption model of CO^+^ ion formation (Schmidt et al. 1995). From the known *A* values, the binding energy of CO molecules adsorbed on a Pt(111) nanofacet as a function of applied electric field can be obtained using Eq. (2), provided, the activation energy of field desorption, *Q*(*F*), is known or can be neglected. The value of *Q*(*F*), which can be derived from ion rate measurements, is in fact the *effective* energy which may be affected by the specifics of the diffusion supply of CO to the desorption sites (CO from the tip-shaft replenishes the CO desorbed from the tip apex). A detailed analysis of the role of the supply (Suchorski et al. 1995; 1996) shows, however, that the effective values are close to the real *Q*(*F*) values, as defined by the scheme in (Fig. 1c). For the present case, the *Q*(*F*) value, as estimated from the measured temperature dependence of the CO^+^ rate, is lower than 0.1 eV, so that the *H*(*F*) − *Q*(*F*) values can be interpreted as the binding energy *H*(*F*) of CO molecules.

**Fig. 3:**
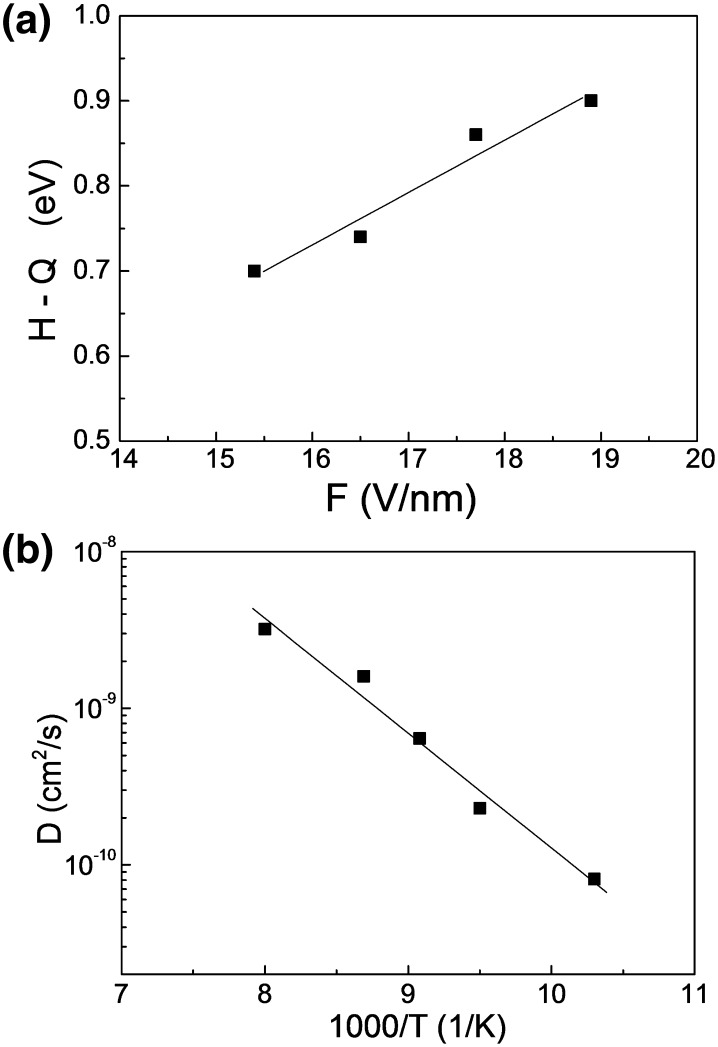
**a** Field-dependent energy term *H*(*F*) − *Q*(*F*) for neutral CO molecules on Pt(111) as a function of the externally applied field strength *F*. **b** Diffusion coefficient D for highly mobile CO species, being the origin of CO^+^ ions emitted from a Pt(111) nanofacet, as a function of the inverse temperature (Arrhenius plot)

Figure 3 displays the values obtained for the binding energy of CO as a function of applied electric field. The *H*(*F*) values range between 0.7 and 0.9 eV for field strengths of 15–19 V/nm. The enhancement of the binding energy by the applied field can be understood on the basis of DFT calculations performed using the nonlocal Backe–Perdev exchange–correlation potential: the applied field stabilizes weakly bound CO molecules via additional field-induced charge transfer, enforcing the Me–C–O orientation (Schmidt et al. 1995). Because the field increases the binding energy, under field-free conditions the detected CO species would certainly exhibit a binding energy of < 0.7 eV. Comparison with adsorption calorimetry (Yeo et al. 1997), adsorption isotherms and thermal desorption results (Ertl et al. 1977), reporting a field-free binding energy of ~ 0.65 eV for Θ = 0.7 ML, reveals that CO^+^ detected in our experiments must originate from weakly bound species (at Θ > 0.7 ML). For strongly bound CO species (at Θ < 0.5 ML) the field-free binding energy is significantly higher (> 1 eV) (Yeo et al. 1997; Ertl et al. 1977), and we can thus exclude such adsorption states as origin of CO^+^ field ions. Weakly bound CO species at high coverages were already reported for other platinum-metal surfaces: e.g. a binding energy of 0.34 eV for Θ > 0.8 was derived in adsorption rate measurements for low temperature CO adsorption on Rh(100) (Medvedev et al. 1997), and a corresponding TPD peak at 100 K was observed for the same system (Peebles et al. 1985).

An evaluation of the diffusivity of CO molecules just before their field desorption corroborates the suggestion of a highly mobile CO layer being the origin of the field desorbed CO^+^ ions. By “opening” the retarder, all CO^+^ ions that have passed the magnetic sector field can be registered and fluctuations of the CO^+^ ion rate can be monitored. Because the fluctuations of number of collected CO^+^ ions are directly related to the surface density fluctuation of CO molecules on the probed surface sites, the diffusivity of the CO species can be evaluated using the *density fluctuation method* as developed by Smoluchowski (1914). The physical basis for such an approach is Onsager’s hypothesis, which states that the microscopic density fluctuation build-up and decay in accord with macroscopic diffusion laws (Onsager 1931). The FIM based techniques are naturally suitable for such approach, since they combine both, the high spatial resolution and the necessary coverage-sensitivity, and allow thus the monitoring of microscopic concentration inhomogeneities in a macroscopically homogeneous layer of adsorbed species [for a survey see e.g. Beben and Suchorski (2003)].

From the registered CO^+^ ion rate fluctuations an *autocorrelation function* can be calculated $A_{\text{N}} \left( t \right)\; = \; \left\langle {\delta N\left( {t \; + \; t^{\prime} } \right)\delta N\left( {t^{\prime}} \right) } \right\rangle _{t^{\prime} }$ where *δN*(*t*) is the fluctuation of *N* at time *t* and $\left\langle {} \right\rangle_{{t^{\prime }}}$ denotes the average over *t′*. From the decay of the autocorrelation function the diffusion coefficient can be directly obtained, following the procedure described in our previous study (Suchorski et al. 1996). Figure 3b shows the results of such an autocorrelation analysis of the rate fluctuations of CO^+^ field ions emitted from a Pt(111) nanofacet as an Arrhenius plot. It is, however, difficult to compare the low activation energy of 0.11 eV, obtained for the present experiments for a stepped Pt-tip surface and an applied field of 15 V/nm, with known field-free data for single crystals [e.g. 0.13 eV for diffusion over terraces and 0.31 eV across steps (Ma et al. 1998)]. Nevertheless, despite the rather qualitative character of the obtained diffusivity and activation energy, the present diffusion experiments provide evidence for a highly mobile CO species being the origin of CO^+^ ions detected by field ion appearance energy measurements.

In summary, the field-stabilization leads to an increased population of highly mobile CO molecules on the Pt tip surface, and thus to the presence of weakly bound CO molecules even at elevated temperatures. In the case of a catalytic reaction (e.g. CO oxidation), this affects the supply of CO into the reaction zone and modifies the dynamics of the reaction itself.

### Extracting Li-adatoms from the Li–O coadsorption layers

Alkali-oxygen coadsorption systems on metal surfaces are interesting both from the fundamental and practical point of view: such effects as alkali metal assisted oxidation of metal surfaces (Driver et al. 1997), or alkali promotion of the ammonia synthesis (Ertl 2003), Fischer–Tropsch reactions (Kiskinowa 1992), oxidation reactions such as ethanol- or CO-oxidation (Avgouropoulos et al. 2006; Pavlenko et al. 2001) are still in the focus of experimental and theoretical studies.

Despite of intensive efforts, the mechanism of catalytic promotion by alkali coadsorption is still not entirely understood and remains a challenging task, because of the diversity of interactions involved in the coadsorption process. Additional complexity in the alkali-oxygen-substrate interaction might arise from the locally modified nanosized surface regions such as steps, kinks, defects etc. Unfortunately, it is difficult to study energetics of such effects, since existing experimental methods, frequently based on thermodesorption, do not provide adsorption energy data with the lateral resolution on a nanoscale. Therefore, the alkali-oxygen coadsorption is a good touch-stone for the laterally resolved measurements based on the appearance energy of field desorbed ions. Since the field desorption of Li from W occurs at RT already at applied fields of 5–8 V/nm whereas more than 25 V/nm are necessary to remove oxygen from the W surface, it is possible to extract Li from the coadsorbed Li/O-layer on tungsten.

**Fig. 4:**
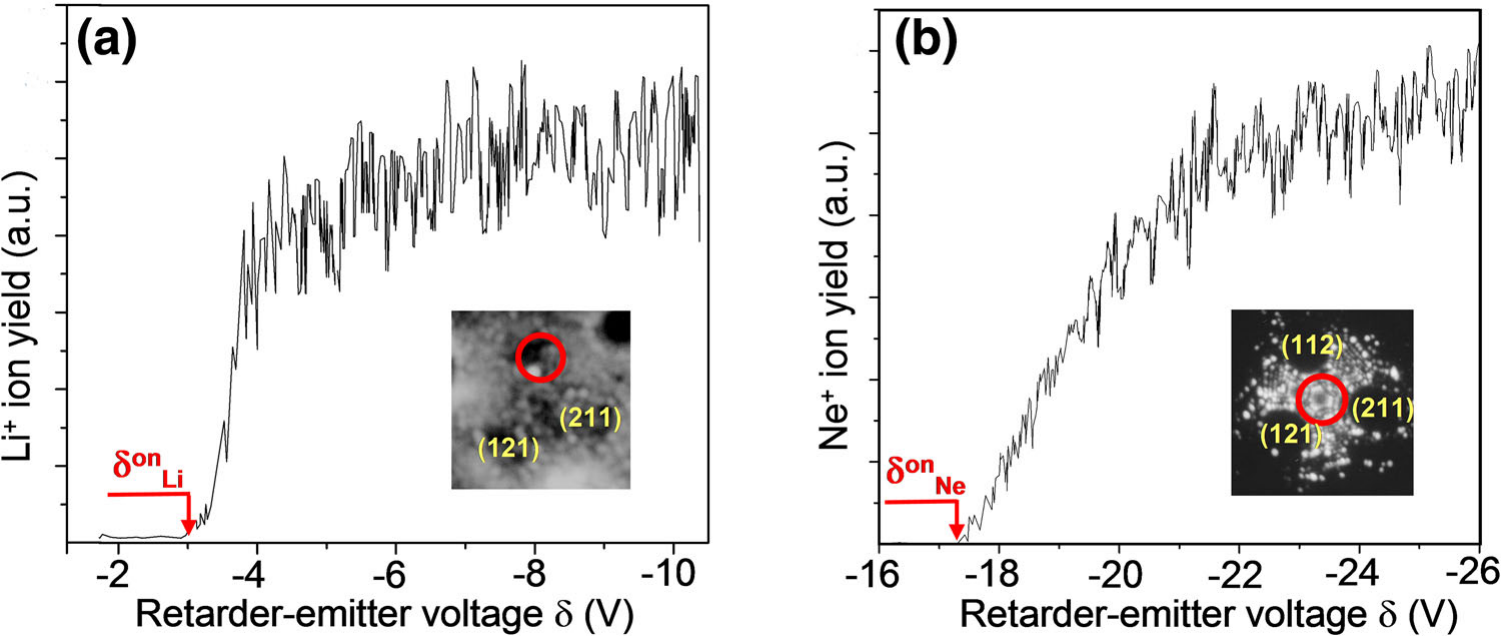
Exctracting Li adatoms from the Li/O coadsorbed layer. **a** Retardation curve for Li^+^ ions emitted from a Li/O coadsorption layer on W(112) facet at 335 K, and at an applied field of 9 V/nm. Lithium and oxygen coverages were 1 ML of Li and 0.5 ML of oxygen, correspondingly. The *inset* shows a Li^+^ FDM image (at 335 K and 13 V/nm) and the position of the probe-hole, **b** the same, but for the Ne^+^ ions emitted from the W(111) facet at 160 K and 35 V/nm. The retardation curve N(δ) was used for the in situ Φ_ret_ calibration. The *inset* shows a Ne^+^ FIM image (at 78 K and 35 V/nm) and the position of the probe-hole

A typical retardation curve for Li^+^ ions emitted from few surface sites on the W(112) apex facet is shown in (Fig. 4a). The corresponding retardation curve for Ne^+^ ions emitted from the W(111) facet (Fig. 4b) was used for the in situ determination of the work function of the retarder, as described above. Retardation curves measured for Li^+^ ions in the applied field range of 5–8 V/nm exhibit extremely steep onsets which are reflected in the extremely narrow energy distribution: the full width at half maximum (FWHM) for the Li^+^ ions (0.35 eV) is much smaller then for Ne^+^ ions (1.7 eV), due to an extremely high degree of spatial localization of the field desorption process. At increasing field strength the onset values *δ* ^*on*^ shift to the right, the appearance energy *A*_Li_ increases. These observations are in good agreement with earlier findings for Li desorbed from a pure Li layer (Suchorski et al. 1995; 1996).

Like in the previous case of CO, the binding energy of Li adatoms coadsorbed with oxygen can be obtained from the appearance energy *A*_Li_ values. However, in the present case of Li, the activation energy of field desorption, *Q*(*F*), is higher than that of CO and cannot be neglected. It can be, however, determined from the rate measurements.

The value of the appearance energy *A*_Li_ = 7.29 eV obtained from the *δ* ^on^ in (Fig. 2a), ionization energy *I*_Li_ = 5.39 eV and activation energy Q = 0.2 eV provide, using the Eq. (2) the value of 2.1 eV for binding energy of Li in a coadsorbed Li/O layer consisting of 1 ML of Li and 0.5 ML of oxygen. The binding energy value appeared to be field independent, at least in the used range 8–13 V/nm, as is also expected from the physical picture of alkali adsorption (Suchorski et al. 1996). It was also possible to study a two-layer configuration: the corresponding binding energy of Li desorbed from the second adsorption layer decreased to 1.8 eV. The measured values are independent of the evaporation order: either the Li was evaporated on the oxygen-precovered surface or oxygen was dosed to Li evaporated on tungsten, also any lithium oxide ions were not detected in our experiments.

Comparison of present values of the binding energy of Li in Li/O-coadsorbed layers with the data obtained for the same Li and O coverage on the single W(112) crystal surface [using the adsorption–desorption equilibrium measurements (Suchorski and Hupalo 2011)], reveal a quantitative agreement of the data obtained locally for few atomic sites on a nm-sized W(112) facet and macroscopic single crystal surface, correspondingly.

## Summary and outlook

In summary, presented exemplary results, obtained by laterally resolved field ion appearance energy spectroscopy, demonstrate, that such kind of spectroscopy, combined with atomically resolving microscopy indeed provides interesting insights into the adsorption processes: the field-stabilization of adsorbed CO leads to an increased population of highly mobile CO molecules on the Pt tip surface, and thus to the presence of weakly bound CO molecules even at elevated temperatures. In the case of catalytic reactions, e.g. CO oxidation, this affects the supply of CO into the reaction opening a route to (partly) bridging the pressure gap. The above measurements can be carried out at *system pressures* up to 10^−4^ mbar. In addition, the field-induced attraction of polarized gas species [the so called “field-compression” effect (Rendulic and Leisch 1980)] enhances the *local pressure* at the tip surface up to 10 times (depending on gas polarizability), thus approaching the 10^−2^ mbar range (which is ca. 10^4^ times higher in pressure than conventional TPD analysis). This increases the local adsorbate coverage and significantly modifies reaction processes. For example, for CO oxidation on a Pt-tip (at 298 K and p_O2_ = 4 × 10^−4^ mbar) in the presence of an applied field of 10–15 V/nm the maximum reaction rate was observed for a CO pressure in the 10^−5^ mbar range. For field-free conditions a 6-times higher CO pressure was required to observe the same maximum on the same Pt-tip (Suchorski et al. 1998).

The apex of a nanotip, as used in present measurements mimics well a single catalytic nanoparticle, exhibiting the same main feature, namely crystallographically differently oriented nanofacets. In contrary to a particle, the surface of the nanotip can be prepared and characterized with atomic resolution (Suchorski and Drachsel 2007). Consequently, using a nanotip as a model catalyst might allow to overcome, at least partially, both the materials and pressure gap. It is thus interesting to compare the above results of CO adsorption on a Pt(111) nanofacet with corresponding high pressure (mbar-range) studies for a Pt(111) single crystal surface using vibrational sum frequency generation (SFG) (Rupprechter et al. 2003). The SFG data for Pt(111) identified a similar dense CO adlayer with ~ 0.7 ML coverage as was detected by high-field studies under vacuum conditions, whereas field ion spectroscopy additionally provided information on the binding energy and diffusivity of weakly bound highly mobile CO species, that may be the active species in CO oxidation reaction.

Novel nanosized materials, such as nanoparticles, nanosheets, and particularly nanorods are suitable specimens for such kind of microspectroscopy, where adsorbed species emitted from e.g. a single nanopore, both from a monolayer or multilayer, might be studied in this way. Moreover, species generated in situ during a catalytic reaction can be analyzed.

## Footnotes

1 http://www.cameca.com/instruments-for-research/atom-probe.aspx.
